# Biogeographical patterns and mechanisms of microbial community assembly that underlie successional biocrusts across northern China

**DOI:** 10.1038/s41522-021-00188-6

**Published:** 2021-02-05

**Authors:** Yuanlong Li, Chunxiang Hu

**Affiliations:** 1 Key Laboratory of Algal Biology, Institute of Hydrobiology, Chinese Academy of Sciences, Wuhan, China; 2 University of Chinese Academy of Sciences, Beijing, China

**Keywords:** Soil microbiology, Microbial ecology

## Abstract

Biocrusts play critical eco-functions in many drylands, however it is challenging to explore their community assembly, particularly within patched successional types and across climate zones. Here, different successional biocrusts (alga, lichen, and moss-dominated biocrusts) were collected across the northern China, and assembly of biocrust microbial communities was investigated by high-throughput sequencing combined with measurements of soil properties and microclimate environments. Bacterial and eukaryotic communities showed that the maximum and minimum community variation occurred across longitude and latitude, respectively. In the regions where all three stages of biocrusts were involved, the highest community difference existed between successional stages, and decreased with distance. The community assembly was generally driven by dispersal limitation, although neutral processes have controlled the eukaryotic community assembly in hyperarid areas. Along the succession, bacterial community had no obvious patterns, but eukaryotic community showed increasing homogeneity, with increased species sorting and decreased dispersal limitation for community assembly. Compared to early successional biocrusts, there were higher microbial mutual exclusions and more complex networks at later stages, with distinct topological features. Correlation analysis further indicated that the balance between deterministic and stochastic processes might be mediated by aridity, salinity, and total phosphorus, although the mediations were opposite for bacteria and eukaryotes.

## Introduction

A major goal in microbial community ecology is to understand assembly processes and their mechanisms across spatial–temporal scales^1,2^. However, it is challenging to study microbial community assembly because of their characteristics of small size, diverse species, rapid dispersal rate, and heterogeneous distribution—differences in the microbial communities several cm apart could exceed those that are several km apart^3^. Moreover, the patterns of microbial community assembly vary depending on the scales across climate zones^4^, and there are obvious successional orders along the temporal sequences in given patchy communities^5–7^. Therefore, it is still a great challenge to explore the community assembly with microbial succession, particularly at a large scale across climate zones^4,8–10^.

Some theories have been developed in the field of microbial community assembly^11,12^. Vellend^13^ combined the habitat filtering driven by differences in adaptive fitness with neutral processes to establish a stochastic–deterministic framework. Furthermore, the quantitative methods for deterministic processes (homogenous selection and heterogeneous selection) and stochastic processes (homogenous dispersal, dispersal limitation, and drift alone) were established by Stegen et al.^14^. It was proposed that selection and neutral processes controlled the microbial communities assembly at different geographic scales^15,16^, and the effects of stochastic processes commonly dominated at larger geographic scales^17–22^. However, regularity of assembly patterns cannot be observed consistently among different microorganisms^16,23–26^ and habitats^19,20,27–30^. It is well recognized that deterministic and stochastic processes played different roles in microbial assembly, which further requires us to explore their respective regulation mechanisms on a given microbial community^4^. It has been found the role of deterministic processes are affected by pH^31,32^, salinity^15,33,34^, organic matters^17,34,35^, aridity index (AI), and mean annual precipitation (MAP)^29,36,37^, while stochastic processes are mainly mediated by AI^38,39^. With growing knowledge about the relative roles of ecological processes changing with spatial–temporal scales^30–32,34^, more microbial assembly studies are focused on the key variables that mediate the balance between them.

Community succession is a sequentially dynamic process integrated over long periods of time and large spatial^40–42^, which provides an opportunity to understand community assembly from a known starting point^34^. There can be reverse succession from mild (e.g., low salinity, neutral pH, and humidity) to severe environments (e.g., high salinity, extreme pH, and drought), and also forward succession (e.g., biofilm and biocrusts) from severe to mild environments. Succession can be characterized by chronological sequence^32,35,40^, or by obvious replacement of dominant taxa^5–7,41,43^. Most studies on community assembly have pointed out the succession is generally associated with chronological community variation, such as coastal salt marsh^34,35^, retreating glacier soils^31,32,44^, and post wildfire disturbed soil^45^. Some reports also showed that pH mediated the community assembly without chronological succession^32^. However, there were only a few studies on the assembly of communities characterized by obviously different dominant taxa and chronological sequences. Particularly, little is known about the interactions of key microorganisms co-occurred in the community assemblies, and the change patterns of community assemblies at geographical scales associated with ecological succession^46^.

Biocrusts are a common life form at the topsoil layer in drylands, which account for 12% of the land surface of the Earth^47^. They play critical ecological functions as they contribute to stabilizing the soil against wind and water erosion and promoting the development of regional environments, also influence soil bacterial composition and diversity in these ecosystems^48^. It can be mainly classified into alga (A), lichen (C), and moss (M)-dominated biocrusts, according to the dominant taxa (cyanobacteria, lichens, or mosses), representing the different successional stages of soil micro-environments/ecosystems^5–7^. Because of their explicit taxonomy at phylum level, easy sampling, and distinguishable phenotypes, biocrusts are widely considered as a model system for soil ecosystem research^5^, however few studies on biocrust community assembly have been conducted. In northern China, there is a wide transect from east to west with changing precipitation and geomorphic features, which provides ideal conditions for biocrust heterogeneous development and the study of their community assembly. In this study, 200 biocrust samples from 50 sites across northern China were collected containing A, C, and M biocrusts. High-throughput sequencing datasets (bacteria and eukaryota) and environmental datasets were simultaneously comparatively analyzed on the basis of succession, longitude, latitude, and MAP to explore the community assembly patterns and regulation mechanisms. We hypothesized that the biogeographical patterns in biocrusts with successional stages would not be influenced solely by spatial factors, and that there would be specific species interactions in a given successional stage of biocrust community.

## Results

### Comparison of community distribution and differences

**Fig. 1:**
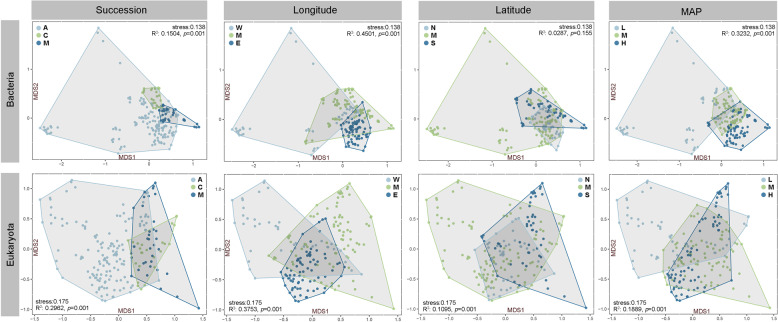
The nonmetric multidimensional scaling of bacterial and eukaryotic communities based on Bray–Curtis distance at operational taxonomic unit level. The NMDS was performed based on succession, longitude, latitude, and mean annual precipitation (MAP), each of which was divided into three groups. Each dot represented a sample, light blue, green, and dark blue corresponded to algae-dominated crust (A), lichen-dominated crust (C), and moss-dominated crust (M) for succession; West (W, E87°–98°), Mid-longitude (M, E98°–109°), East (E, E109°–120°) for longitude; North (N, N42°–45.5°), Mid-latitude (M, N38.5°–42°), and South (S, N35°–38.5°) for latitude; Low-MAP (L, 0–150 mm), Mid-MAP (M, 150–300 mm), and High-MAP (H, 300–450 mm) for MAP, respectively. ANOSIM analysis and permutation test (999) were used to test for significant differences among groups.

The orders of presentation and analysis that we defined for this study were according to sample successional stages from A to C to M, longitude location from West to Mid-longitude to East, latitude location from North to Mid-latitude to South, and MAP from Low to Middle to High, characterized by the gradual changes from severe to medium to mild environment. Non-metric multidimensional scaling (NMDS) ordination was used to visualize Bray–Curtis dissimilarities of communities. The difference and significance among the three groups for each study angle were tested by analysis of similarities (ANOSIM, permutation test = 999). The results indicated that bacterial and eukaryotic communities were significantly different for all study angles except for bacterial communities on latitude angle (*p* > 0.05, Fig. 1). For bacteria, the community differences were in the order of longitude > MAP > succession > latitude, while for the eukaryotic community they were in the order of longitude > succession > MAP > latitude.

For bacterial community variations among the three groups within each study angle (Fig. 1), the variation in succession (ANOSIM: *R*^2^ = 0.1504, *p* = 0.001; betadisper: *F* = 32.993, *p* = 0.001) was the greatest at the early successional stage (i.e., A biocrust), next at late stage (i.e., M biocrust) and the smallest at middle stage (i.e., C biocrust); for longitude (ANOSIM: *R*^2^ = 0.4501, *p* = 0.001; betadisper: F = 46.418, *p* = 0.001), the variation decreased gradually from West to Mid-longitude to East and for latitude (ANOSIM: *R*^2^ = 0.0287, *p* = 0.155; betadisper: *F* = 30.071, *p* = 0.001) the order was Mid-latitude > South > North; for MAP (ANOSIM: *R*^2^ = 0.3232, *p* = 0.001; betadisper: *F* = 63.876, *p* = 0.001), the greatest variation was observed for Low followed by High, with minimal differences in Middle precipitation groups. For the eukaryotic community, the order of variation in succession (ANOSIM: *R*^2^ = 0.2962, *p* = 0.001; betadisper: *F* = 15.125, *p* = 0.001) was the same as that of the bacterial community; for longitude (ANOSIM: R^2^ = 0.3753, *p* = 0.001; betadisper: *F* = 2.925, *p* = 0.072), the divergent communities were most in West and East groups, while convergent communities were most in the Mid-longitude group; for latitude (ANOSIM: *R*^2^ = 0.1095, *p* = 0.001, betadisper: *F* = 18.637, *p* = 0.001), there was great variation in the South and Mid-latitude groups, and relatively small variation in the North group; for MAP (ANOSIM: *R*^2^ = 0.1889, *p* = 0.001; betadisper: *F* = 3.759, *p* = 0.031), the community differences among the three groups were similar.

Linear regression analysis was used to find environmental variables that changed most dramatically among the three groups of the four study angles (Supplementary Table 1); these were thickness (TH.) and oxidation-reduction potential (ORP) for succession, wind speed (WS) and altitude (Alt.) for latitude, and MAP and AI for both longitude and MAP.

**Fig. 2:**
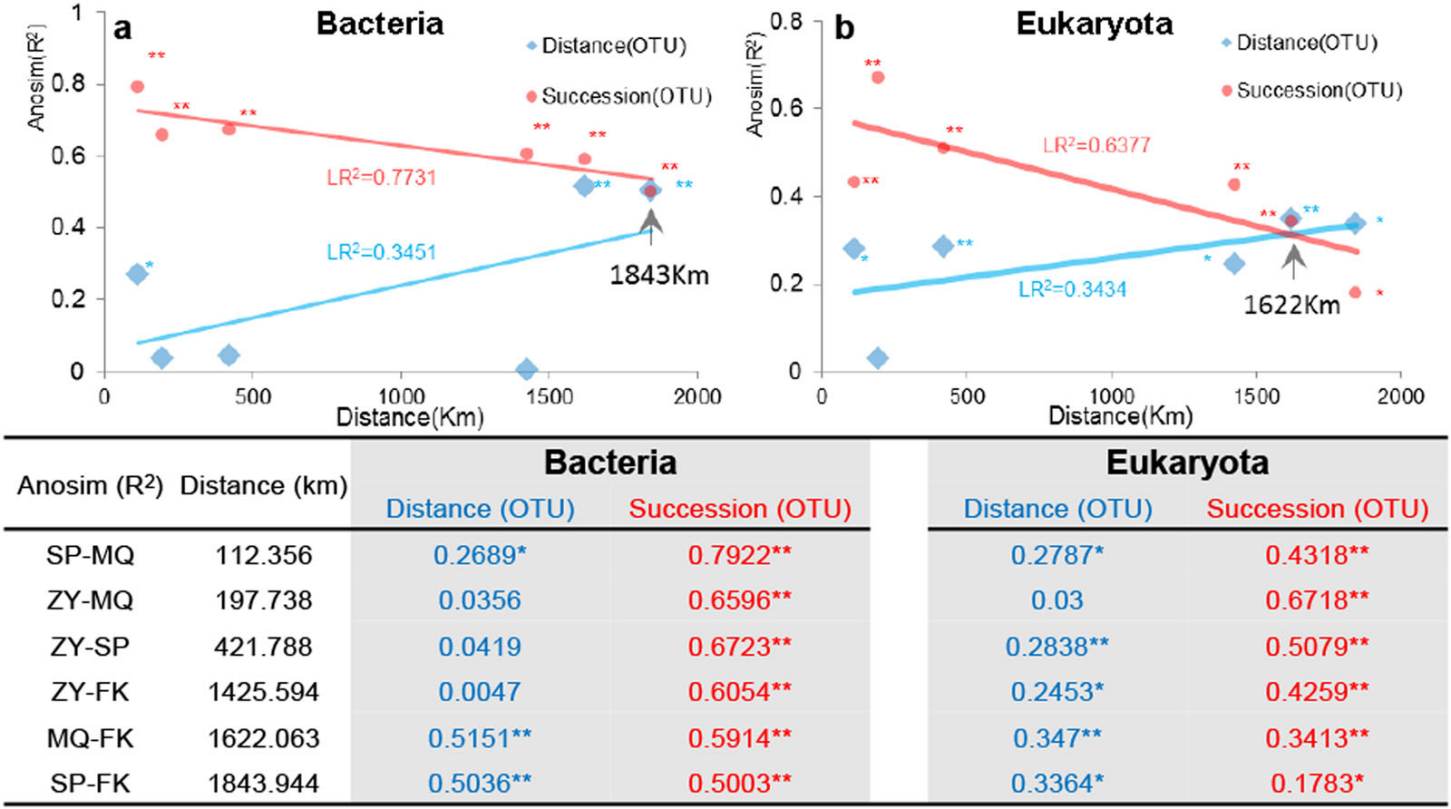
Community differences varied with distance in succession and geography at regional scales. Sample sites contained FK, ZY, MQ, and SP (see Fig. 5). Bacterial (**a**) and eukaryotic (**b**) community differences were compared based on Bray–Curtis distance at operational taxonomic unit level on succession (red line) and distance (blue line). Linear regression (LR) was performed between ANOSIM (*R*^2^, permutation test = 999, *<0.05, **<0.01) and distance (km).

To exclude the influence of the unequal meridional span of the three successional stages of biocrusts (A biocrusts ~2500 km, M biocrusts ~1700 km, and C biocrusts ~1300 km) on microbial community differences, sample sites with three types of biocrusts were selected for comparative analysis at regional scale. At regional scale (Supplementary Table 2), the bacterial community differences were in the order of succession > MAP > longitude > latitude, while the eukaryotic community differences were in the order of succession > longitude > MAP > latitude. We found the maximum difference among successional stages for both bacterial and eukaryotic communities. Considering that there are community differences among geographic position and succession stages, the linear regression analysis of community difference with geographical distance showed that microbial community difference among sample sites increased with distance regardless of succession factor, and the community difference decreased with distance when only taking succession factor into consideration (Fig. 2). The opposing direction of the change in microbial community difference influenced by distance and succession factor resulted in similar result at a distance of 1843 km and 1622 km for bacterial and eukaryotic communities, respectively.

### The correlation between community structure and environmental variables

**Fig. 3:**
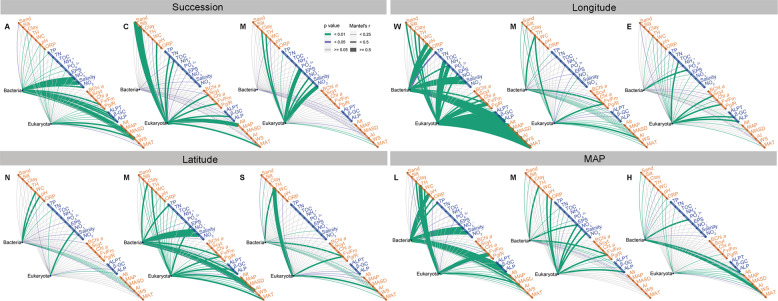
Correlations between environmental factors and microbiome community at phylum level by Mantel test. The environmental factors were divided into five categories: soil properties (sand, silt, clay, thickness (TH.), water content (WC), pH, oxidation-reduction potential (ORP)), nutrition and ions (total phosphorus (TP), total nitrogen (TN), total organic carbon (TOC), NH_4_^+^, PO_4_^3+^, extracellular polysaccharide (EPS), NO_3_^−^, salinity, NO_2_^−^), photosynthesis and pigments (bacteriochlorophyll *a* (BChl *a*), scytonemin (Scyt.), chlorophyll *a* (Chl *a*), variable fluorescence/maximal fluorescence (Fv/Fm), gross photosynthesis/respiration (Pg/R), extracellular enzyme activity (soil alkaline protease (ALPT), and soil-β-glucosidase (β-GC), soil alkaline phosphatase (ALP)), and microclimate (altitude (Alt.), mean annual precipitation (MAP), mean annual sunshine duration (MASD), aridity index (AI), windspeed (WS), mean annual temperature (MAT)), separated by yellow and blue. The variables that were most strongly correlated with bacterial and eukaryotic communities were salinity (*r* = 0.81) and MAP (*r* = 0.32) in A crust; scyt. (*r* = 0.24) and silt (*r* = 0.81) in C crust; WC (*r* = 0.24) and NO_2_^−^ (*r* = 0.65) in M crust; salinity (*r* = 0.81) and Fv/Fm (*r* = 0.75) in West (W); TH. (*r* = 0.47) and Fv/Fm (*r* = 0.43) in Mid-longitude (M); PO_4_^3+^ (*r* = 0.28) and ALP (*r* = 0.34) in East (E); WC (*r* = 0.30) and NO_3_^−^ (*r* = 0.45) in North (N); salinity (*r* = 0.81) and Fv/Fm (*r* = 0.45) in Mid-latitude (M); TH. (*r* = 0.39) and TH. (*r* = 0.54) in South (S); salinity (*r* = 0.80) and TH. (*r* = 0.58) in Low-precipitation (L); TH. (*r* = 0.47) and NO_3_^−^ (*r* = 0.36) in Mid-precipitation (M); Alt. (*r* = 0.39) and AI (*r* = 0.25) in High precipitation (H).

To explore the correlation between community structure and environmental variables, Mantel test analysis was performed with community matrices at phylum level and environmental factors on three gradients of the four study angles (Fig. 3). For succession, we found that the bacterial community at the early successional stage was strongly correlated with salinity, extracellular enzyme activity and macroclimate, while the eukaryotic community was strongly correlated to macroclimate variables (MAP and AI) alone; at the middle successional stage, only the eukaryotic community was intensely correlated with soil texture and macroclimate (MAP), and with trophic ions (PO_4_^3+^ and NO_2_^−^) at the late stage. For longitude, the number of environmental variables were strongly correlated with the microbial community decreased from West to Mid-longitude to East. For latitude, the bacterial community was obviously correlated with water content (WC) and pH, while the eukaryotic community was obviously correlated with NO_3_^−^ in the North group. Meanwhile, both bacteria and eukaryotes were strongly correlated with TH., variable fluorescence/maximal fluorescence (Fv/Fm), and so on in the South. Similar to the changes in longitude from West to Mid-longitude to East, the number of variables strongly correlated with the microbial community decreased with precipitation. In conclusion, the variables intensely correlated with microbial communities for the four study angles were obviously different. Specifically, there were many variables correlated with the microbial communities in the West, Mid-latitude, and Low MAP groups, and limited variables in the East, South and North latitude, and High MAP groups.

### Ecological processes of community assembly

We quantified the relative roles of ecological processes affecting community assembly for the four study angles (Table 1 and Supplementary Table 3). Community assembly of biocrusts was mostly dominated by dispersal limitation coupled with drift, presenting as a heterogeneous aggregation, except in the middle successional stage and in the West where eukaryotic community assembly was greatly affected by drift alone. Moreover, the proportion of species sorting (mainly heterogeneous selection) in the bacterial community was much higher than that in the eukaryotic community (mainly homogeneous selection). For succession, the relative quantification of ecological processes driving bacterial assembly showed irregular change along the succession, except the extremum tended to appear in the middle stage (Table 1 and Supplementary Table 3). However, the contribution of homogeneous selection, homogeneous dispersal, and species sorting increased, and that of dispersal limitation decreased, with succession of the eukaryotic community. From West to East, the degree of bacterial community homogenization declined and eukaryotic community heterogenization increased (Table 1 and Supplementary Table 3). Meanwhile, species sorting had the greatest impact on the bacterial community in the West. The role of dispersal in the assembly of the eukaryotic community increased, and the role of drift decreased for longitude. For latitude, there was an obvious decline in heterogeneous selection and an increase in homogeneous selection in the bacterial community from North to South (Table 1 and Supplementary Table 3) that led to the species sorting decline, which confirmed the major role of heterogeneous selection in species sorting. With the increase in precipitation, drift and homogeneous dispersal became less important in integral community assembly (Table 1 and Supplementary Table 3). Conversely, the impact of heterogenous selection increased for both bacterial and eukaryotic communities. Homogeneous selection decreased in importance of the bacterial community, while dispersal limitation increased in importance of the eukaryotic community. Species sorting had the greatest impact on the eukaryotic community in the Middle precipitation group.

**Table 1:** Quantification of the relative roles of ecological processes in bacterial and eukaryotic community assembly. Values indicate the percentage of community turnover associated with each process.

	Succession			Longitude			Latitude			MAP		
	A	C	M	W	M	E	N	M	S	L	M	H
Bacteria												
Homogeneous selection(%)	1.13	3.99	0	11.81	1.29	0.2	1.8	1.83	2.26	8.31	0.74	0.27
Heterogeneous selection(%)	23.75	1.09	21.25	28.5	15.26	18.44	26.56	22.47	18.72	15.72	7.03	30.22
Drift alone(%)	12.98	48.19	13.1	13.74	13.25	30.85	16.51	9.59	14.04	14.74	11.96	7.91
Homogeneous disper(%)	1.38	7.97	4.47	6.55	2.82	1.12	3.92	1.64	5.06	5.52	2.36	1.84
Disper limit coupled drift(%)	60.63	38.77	61.18	39.41	67.39	49.38	51.22	64.47	59.92	55.71	77.91	59.77
Eukaryota												
Homogeneous selection(%)	4.42	7.69	34.44	5.91	18.95	4.04	8.04	4.91	19.92	5.82	11.91	6.86
Heterogeneous selection(%)	0.03	0	0	0.26	0.03	0	0	0.06	0	0.23	0	0
Drift alone(%)	21.48	65.57	9.68	48.01	22.52	20.46	37.04	19.24	25.89	35.17	25.92	12.97
Homogeneous disper(%)	1.05	2.93	55.87	2.44	3.95	4.5	5.19	1.48	4.08	2.72	2.68	2.31
Disper limit coupled drift(%)	73.02	23.81	0	43.39	54.55	71	49.74	74.32	50.11	56.05	59.49	77.86

### Species co-occurrence patterns

**Fig. 4:**
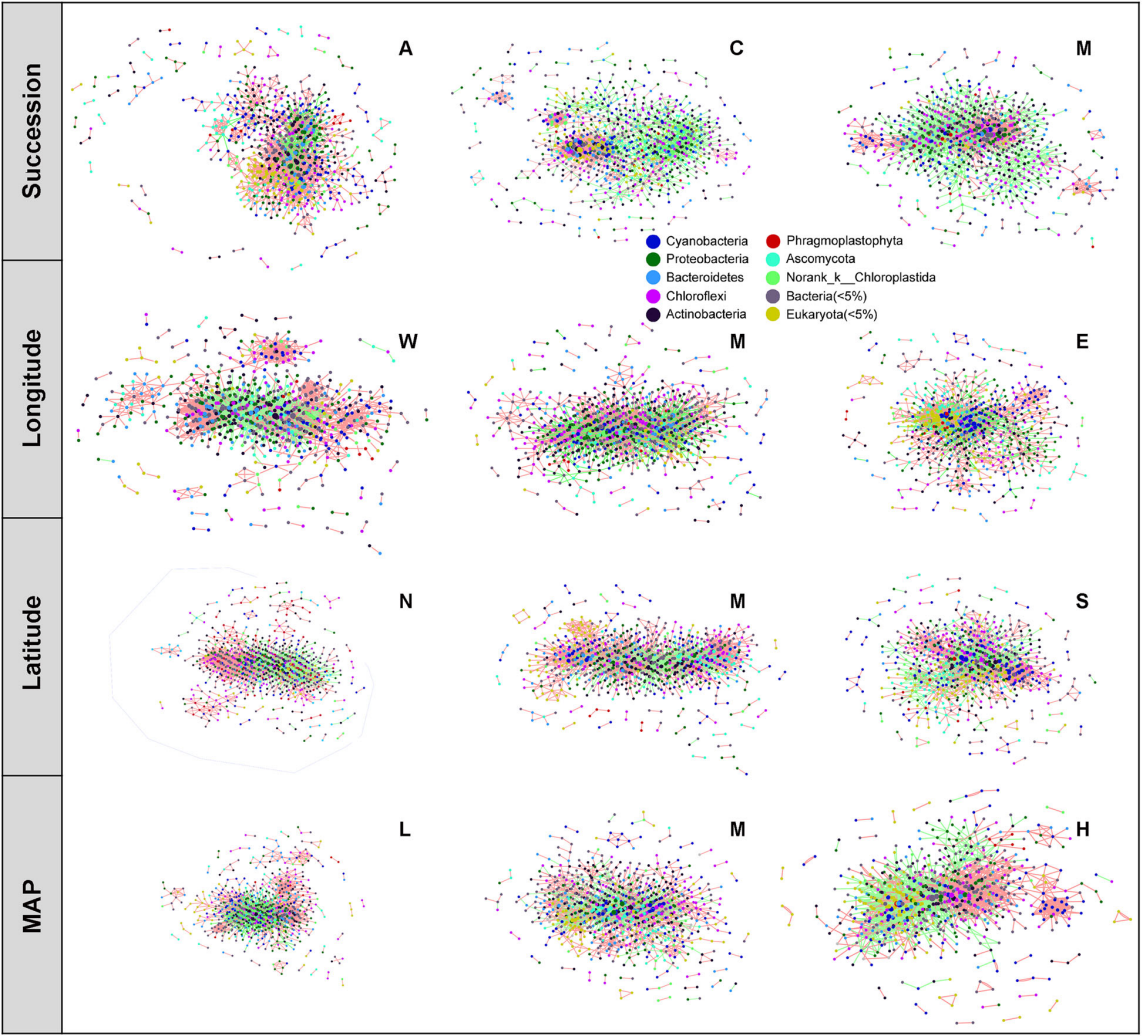
Co-occurrence network of microbiomes based on succession, longitude, latitude, and mean annual precipitation (MAP) study angles. Each study angles was partitioned into three groups, algae-dominated crust (A), lichen-dominated crust (C), and moss-dominated crust (M) for succession; West (W), Mid-longitude (M), East (E) for longitude; North (N), Mid-latitude (M), and South (S) for latitude; Low-MAP (L), Mid-MAP (M), and High-MAP (H) for MAP, respectively. The size of each node was proportional to the degree of the operational taxonomic units. Node color was based on phylum taxa. The connection (edge) colored with red and green represented coexistence and mutual exclusion, respectively. See Table 2 and Supplementary Table 4 for more detailed topological characteristics.

To understand the relationship between species co-occurrence patterns and microbial assembly, we inferred a metacommunity co-occurrence network (Fig. 4) for the four study angles using Operational taxonomic units (OTUs) with 99% cumulative abundance of bacterial and eukaryotic communities and captured associations based on Spearman correlation with a threshold of 0.7. Subsequently, we calculated a set of topological features (Table 2).

**Table 2:** The topological characteristics of the co-occurrence network.

	Succession			Longitude			Latitude			MAP		
	*A*	*C*	*M*	*W*	*M*	*E*	*N*	*M*	*S*	*L*	*M*	*H*
Num. edge/Num. node^a^	2.96	4.04	4.66	6.02	3.95	4.198	5.05	4.09	4.88	6.27	3.33	5.67
The proportion of coexistence edge (%)	87.85	68.80	66.25	62.29	61.44	59.75	56.28	73.19	53.90	62.75	58.49	56.34
The proportion of mutualexclusion edge (%)	12.15	31.20	33.75	37.71	38.56	40.25	43.72	26.81	46.10	37.25	41.51	43.66
Max. degree	45	61	74	163	71	74	87	77	81	175	57	132
Clustering coefficient^b^	0.34	0.27	0.22	0.34	0.29	0.34	0.29	0.32	0.34	0.36	0.31	0.33
Connected components^c^	45	44	31	34	37	31	35	34	36	30	29	26
Network centralization^d^	0.051	0.062	0.079	0.174	0.069	0.095	0.086	0.083	0.097	0.200	0.061	0.159
Network density	0.008	0.009	0.011	0.014	0.009	0.012	0.011	0.010	0.013	0.015	0.008	0.015
Network heterogeneity^e^	1.20	1.45	1.50	1.33	1.27	1.49	1.28	1.27	1.36	1.4	1.29	1.41
Average degree^f^	5.93	8.10	9.33	12.05	7.90	8.40	10.12	8.18	9.78	12.55	6.68	11.35
Average path length	4.55	5.02	4.61	4.24	4.09	4.31	4.06	4.02	4.12	3.41	4.49	4.01
The proportion of Eukaryota node (%)	23.57	18.04	9.30	14.71	13.22	31.84	15.11	20.57	28.15	16.77	23.35	25.00
The proportion of Bacteria node (%)	76.43	81.96	90.70	85.29	86.78	68.16	84.89	79.43	71.85	83.23	76.65	75.00

^a^Average connection flux per node.

^b^Average value of each node clustering coefficient in network, which indicateed nodes were embedded in their neighborhood and interconnected together.

^c^The number of subgraphs were not connected with other subgraphs, indicating the modularity.

^d^Measurement of the tendency towards the center of the network.

^e^Measurement of the ability to form a central node in the network.

^f^The average degree of each node, which showed how many connections (on average) each node had in the network.

Co-occurrence species exhibited different patterns for the four study angles (Fig. 4, Supplementary Table 4). There were growing bacterial nodes and declining eukaryotic nodes with succession orders. However, the pattern reversed from West to East and from Low to High precipitation. In the central large cluster, most coexisting rare microorganisms including bacteria and eukaryota were contained in A biocrusts; both coexisting rare microorganisms and abundant mutual exclusion microorganisms including bacteria and eukaryota were contained in C biocrusts (Fig. 4, Supplementary Table 4). In M biocrust, the main co-occurring species were Proteobacteria and Chloroflexi (Anaerolineales). For longitude, rare bacteria mainly dominated in the West group, while Cyanobacteria and rare eukaryota dominated in the East group. For latitude, there were mostly Proteobacteria and Chloroflexi (Thermomicrobia) in the North group and Cyanobacteria in the South group. For MAP, rare bacteria mainly dominated in low precipitation, while the highest numbers of co-occurring species in High precipitation were identified as Proteobacteria and Chloroflexi (Thermomicrobia).

In terms of co-occurrence relationships, the network pattern of the four study angles presented a high degree of nodes in the central cluster and a low degree of nodes in peripheral positions. First, except for the Mid-latitude, the proportion of coexisting species decreased and the proportion of mutual exclusion increased following the group order we defined in the four study angles respectively. Second, the indicators of the average connectivity flux of nodes, max. degree, network centralization, network density, average degree of nodes, and network heterogeneity increased, and those of the network clustering coefficient, and connected components decreased with succession. Nevertheless, the number of connected components changed inversely with precipitation, and only the clustering coefficient increased from North to South. Third, there were coexisting clustered modules for all successional stages, the West, East, South, Low, and High precipitation groups. Fourth, the effect of dispersal limitation on the eukaryotic community was positively correlated with the clustering coefficient and on the bacteria community was negatively correlated with average connectivity flux of nodes, network centralization, and density (Supplementary Table 5).

### Environmental variables that mediated the balance between deterministic and stochastic processes

Correlation analysis between the environmental variables and the relative proportion of species sorting was conducted to determine the environmental variables that mediated ecological processes (Supplementary Table 6). The results generally demonstrated opposite covariant directions according to the relationships between the relative proportion of species sorting and bacterial and eukaryotic communities. AI, salinity, and total phosphorus (TP) were related to both bacterial and eukaryotic communities. Furthermore, pH, ORP, MAP, and mean annual sunshine duration (MASD) were only related to species sorting in bacteria, WC, NH_4_^+^, and PO_4_^3+^ were only related to species sorting in eukaryotes.

## Discussion

Because of the complex environmental characteristics contained in the spatial scale, it is difficult to understand the mechanism behind microbial community assembly in different biogeographic distributions^4,8–10^. Biocrusts are widely distributed in drylands all over the world with community dissimilarity not only between patches cm apart^7^, but also between successional stages. In the present study, we observed that both bacterial and eukaryotic communities showed the greatest and the least differences along longitudinal and latitudinal gradients. Successional stages and MAP had the second greatest differences for bacterial and eukaryotic communities, respectively. First, these results suggested that the meridional distance (~2500 km) was still the primary factor for the differences in heterogeneous communities^49^. The biogeographic pattern responded to precipitation less than to longitude, although precipitation changed most dramatically along longitude (Supplementary Table 1). Second, the spatial factor not only included the distance but also the topographical features^50^ such as mountain terrain^10^. Third, when excluding the influence of the unequal meridional span of the three successional stages, the difference in microbial communities among successional stages was greatest at the regional scale (Supplementary Table 2). This result was inconsistent with that at continental scale and showed that the impact of succession still existed at regional scale^21,51^. This result demonstrated that the succession community had a universal distance-decay pattern (Fig. 2). The spatial factors including topographical features, soil texture, macroclimate, wind direction (mostly northwest wind), and other environmental characteristics^49–52^ resulted in different regional species pools that should not be ignored^9^. However, the same species had multiple possibilities to be sorted into different successional biocrusts which resulted in the difference of community decreased with distance for bacteria and eukaryota if succession factors were only considered (Fig. 2). Given that these two changes intersected at a certain distance, minimizing the difference from sampling at different spatial scales is still a problem that needs to pay attention in large-scale microbial ecological research^46^, especially for heterogeneous microbial communities without phenotypic reference.

Biocrust succession is classified based on the different dominant cryptogamous plants and productivity^7,43^, which has been distinguished from the temporal-based succession^32,35,40^. Community assembly is dominated by stochastic processes at all successional stages in biocrust, unlike that in salt marshes and after fire disturbances, which are dominated by stochastic processes only in early succession^35,45^. First, we thought that assembly patterns driven by varying ecological processes would mainly be subject to the available energy source or the trophic type of abundant taxa in ecosystems^53^. Therefore, according to the classification criterion of biocrust succession, it was reasonable to get similar community assembly patterns among biocrust successional stages because the dominant microorganisms are all primary producers and undeniably driven by light energy^7,54^. Second, with succession, the eukaryotic community showed a drastic trend of increasing homogeneity and decreasing heterogeneity. Additionally, the relative role of species sorting increased in the eukaryotic community until it exceeded that in the bacterial community at late succession (Table 1 and Supplementary Table 3). These results suggested that environmental filtering mainly affected eukaryotes, and the high heterogeneity in the late stage^6,7,40,42^ mainly resulted from non-microeukaryota. Third, the strongly correlated variables varied with successional stages, especially those related to the eukaryotic community (i.e., macroclimate (MAP, AI) in A biocrust, soil texture, and macroclimate variables (MAP) in C biocrust, trophic ions (PO_4_^3+^, NO_2_^–^) in M biocrust; Fig. 3). These results differed from the situation in retreating glacier soil which was always driven by pH^32^ and in salt marsh that was driven by salinity in the early stage^35^, while they were consistent with the situation in the terrestrial macroecosystem^55^ and late primary succession with limited resources in many habitats^56^. Fourth, the microbial community structure driven by convergent ecological processes was correlated with divergent environments in biocrusts. The proportion of deterministic processes increased with succession for the eukaryotic community, resembling the bacterial community in salt marsh^35^. Moreover, the assembly patterns dominated by stochastic processes in biocrusts with neutral pH were similar to those in retreating glacier soils with varying pH^32^. Taken together, these results showed that the assembly pattern was the final outcome of that driven or mediated by diverse mechanisms; the results also illustrated that the deterministic processes dominated the direction of succession in biocrust.

The succession stages of A, C, and M biocrusts corresponded to the severe, medium, and mild environmental gradients^6,7,43^, as with the other three study angles following the order that we defined. The community assembly of biocrusts was mostly dominated by dispersal limitation coupled to drift for the four study angles, which was consistent with most results in desert habitats^17,18,38,57^. Besides, the role of drift was quite similar for all four study angles and was shown to be mediated by AI^38^ and to balance the fungal abundance in the eukaryotic community^15,27,30^. Additionally, it was speculated that habitat differences contributed to the decreasing proportion of heterogenous selection in the bacterial community from North to South, contrary to cropland^30^. The results also demonstrated that the effect of species sorting on bacterial assembly was greater in the severe environment than in the mild environment for most study angles except MAP, which was the opposite to that of the eukaryotic community for most study angles except longitude (Supplementary Table 3), suggesting that environmental filtering selected more bacteria in the severe environment than in the mild environment and that the opposite relationship existed for eukaryotes. The effect of dispersal on the bacterial community did not differ much between A and M biocrusts, while it was weaker for M biocrusts than for A biocrusts on the eukaryotic community. This explained the high bacterial heterogeneity^6,7,40,42^ and domination by eukaryotes^7,43^ in the late successional stage. Moreover, it also explained the decrease in the dissimilarity of the microbial community with the increase in geographical distance when only succession was considered (Fig. 2). Finally, we concluded that assembly patterns for the different study angles were probably related to the adaptation mechanism of dominant microorganisms^7,43^ and the ability to modify the environments^6^.

The correlations between microbial communities and environmental variables for the four study angles might also explain the different assembly patterns. Despite the possibilities of microbial dormancy, a considerable number of correlations existed between environments and microbial communities in West, Low-precipitation, and Mid-latitude groups where habitats were hyperhaline or drought-affected because of their advantages in adapting to environmental stress^46^. Conversely, a limited number of correlations were obtained in East, High-precipitation, North, and South groups where habitats were moist or in low altitude (Fig. 3). After comprehensive comparison, we speculated that the patterns of microbial assembly were closely correlated to their representative variables for the four study angles. In this regard, previous studies have shown that AI^36^, precipitation pattern^37^, and available water^58^ could significantly restrain the cyanobacteria abundance in biocrusts; ORP played an important role in successional community structure^28,59^; WS was the main stress environmental factor^60^ for biocrust formation and also an important variable^61^ that affected the surface microbial communities; the Alt. and TH. affected the biocrust community structure by impacting on microclimate^62^ and light^6^, respectively. The environmental variables mentioned above were consistent with our results for the four study angles (Supplementary Table 1).

Species competition is a non-negligible part of the co-occurrence theory and an important driving force to assemble microorganisms. However, we have limited understanding of its variation in community assembly^63^. The proportion of mutual exclusion relationships increased while the proportion of coexistence decreased with environmental characteristics from severe to mild for the four study angles (Table 2), which was another illustration of the increased competition caused by the improving environment^63^ and the enhanced role of deterministic processes caused by biotic factors. The coexistence relationships were higher than those of mutual exclusion in this study, which was opposite to that in river sediments^20^. The average path length (ave. 6.68–12.55) in biocrusts was shorter than that in rice soil indicating weak connections^30^ in the biocrust environment. Briefly, the microecosystem of biocrusts was not as mature as that of sediment^20^, presenting a pattern of high coexistence and low competition.

The topological features of the network were used to reveal co-occurrence patterns^64–66^. The co-occurrence patterns of the four study angles were generally similar but not identical. First, we found more bacteria than eukaryota in our co-occurrence network^20^, which was similar to the situation in sediment with co-occurrence of few fungi. Meanwhile, the network heterogeneity and average degree of each node increased with succession, which implied an intricate network at late successional stage. From this point of view, the co-occurrence pattern along the succession in biocrust was opposite to that in salt marshes^35^. Second, the gradual change of average degree, clustering coefficient, connected components, and network centralization with succession implied a co-occurrence pattern characterized by low connection flux, compacted clustering, and more modularity around a weaker center that was mostly composed of rare taxa at the early stage; this network was converted to one characterized by high connection flux, less compacted clustering, and less modularity around a stronger center composed of abundant taxa at the late stage (Table 2). In terms of latitude, we deduced from the increasing network clustering coefficient, which was the only regularly changing feature, that might be passive clustering affected by wind. Third, based on the relationships of assembly and co-occurrence pattern^38,67,68^, the obscure boundary of modules in the co-occurrence network was a significant characteristic of community assembly dominated by stochastic processes, which was confirmed by our results. Conversely, because we found more modules in the severe environment than in the mild environment, this suggested^64,68^ that the clustering of modules was facilitated by various environmental factors (Table 2).

We found that bacteria were present at higher abundance than eukaryotes in the co-occurrence network (Table 2) and bacterial community assembly was more dominated by deterministic processes than that of eukaryotes (Supplementary Table 3). Considering that the desert environment included many stress factors and the relationships between the relative proportion of species sorting and AI, salinity, and TP were opposite for the bacterial and eukaryotic communities, respectively (Supplementary Table 6). We proposed that the environmental variables affecting the deterministic process could be the key to balance ecological processes. It has been shown that AI^36^ and salinity^15,33,34^ were common variables regulating microbial community structure especially in arid areas and TP^69^ was the restrictive variable for primary production in the terrestrial biosphere. Other studies also showed that pH^31,32^, ORP^28,59^, MAP^37^, and MASD^17,54,61^ were related to bacterial species sorting, and WC^6^ and trophic ions (NH_4_^+^, PO_4_^3+^)^56^, were related to eukaryotic species sorting. Both of them had substantial impact on microbial succession. Under the consensus that the biogeographic pattern was formed by ecological processes^16^, the assembly of bacterial and eukaryotic communities in biocrusts was mainly affected by stochastic processes rather than by deterministic processes. Given the above, we proposed that the deterministic and stochastic processes of biocrust assembly might be mediated by varying degrees of different variables and opposing influences of AI, salinity, and TP. In this way, the assembly of biocrusts community had characteristics of both microorganisms and terrestrial plants.

Drought, as a deterministic factor^36,37^, was also a key variable regulating the stochastic process^38^. The microbial assembly in biocrust was dominated by dispersal limitation as expected and in line with the windy and drought-affected desert background^17,18,38,57^. The fact that eukaryotic community assembly was mainly driven by neutral processes and bacterial community assembly was more influenced by species sorting (~40%) in the West group manifested the effect of environmental stress. Additionally, the high proportion of species sorting and homogeneous assembly in the eukaryotic community also indicated that the eukaryotes required suitable environments^25,26^. The microorganisms in the arid species pool all have niche advantage. While, there were differences in fitness cause of environmental stress. We considered this is a reason why the bacterial/eukaryotic community assembly was dominated by dispersal limitation coupled to drift.

Because of the inconsistent regularity of assembly patterns observed among different microorganisms^16,23–26^ and habitats^19,20,27–30^, we considered that: 1) their community assembly may be mediated in the opposite way by the same factors; 2) the large-scale investigation conducted here allowed the recognition of the role of spatial factors^51^, and presented a spartial pattern^46^. However, this framework was not conducive to finding the key variables driving ecological processes under different assembly patterns; 3) we suggested that more than three comparable groups should be designed along the habitat gradients to find the variables mediating community assembly^39^ for non-model samples and multiangle analysis should be carried out.

In conclusion, 1) both bacterial and eukaryotic communities showed the greatest and the smallest differences along gradients of longitude and latitude in the arid and semiarid areas of northern China (meridional distance 3470 km). The second greatest community difference of bacteria and eukaryota was respectively found at succession angle characterized by heterogeneity at distances of several cm and at MAP angle characterized by dramatic longitudinal change. However, we found the maximum difference among successional stages for both bacterial and eukaryotic communities at a regional scale. 2) The community assembly of biocrusts was mostly dominated by dispersal limitation coupled to drift. In the most drought-affected areas, eukaryotic community assembly was mainly driven by neutral processes and bacterial community assembly was more influenced by species sorting. In the Mid-latitude and Mid-precipitation areas, the highest proportion of species sorting and homogeneous assembly were found for the eukaryotic communities. 3) An interesting characteristic of microbial assembly associated with biocrust succession was that eukaryotic communities showed increasing homogeneity, with increased species sorting and decreased dispersal limitation for their assembly patterns. Furthermore, the variables mainly affecting eukaryotic community assembly still depended on biocrust successional stages (Fig. 3), such as macroclimate variables (MAP and AI) in A biocrust, soil texture, and macroclimate variables (MAP) in C biocrust, and trophic ions (PO_4_^3+^, NO_2_^–^) in M biocrust. These suggested that the different dominant microorganisms might have varieties of adaptation mechanisms and abilities to modify the environments. 4) Weak competition and strong coexistence were a typical co-occurrence pattern in the early successional stages. The proportion of competition increased and the co-occurrence network became more intricate along with the succession. 5) The co-occurrence pattern was characterized by low connection flux, compacted clustering, and high modularity around a weak center mostly composed of rare taxa in the early successional stage. On contrast, in the late successional stage, the co-occurrence pattern was characterized by high connection flux, less compacted clustering, and less modularity around a stronger center composed of abundant taxa. 6) Microbial community assembly in biocrust could be mediated by different variables, and our results suggested that AI, salinity, and TP presented opposite influences on bacterial and eukaryotic communities.

## Method

### Soil sampling and data collection

**Fig. 5:**
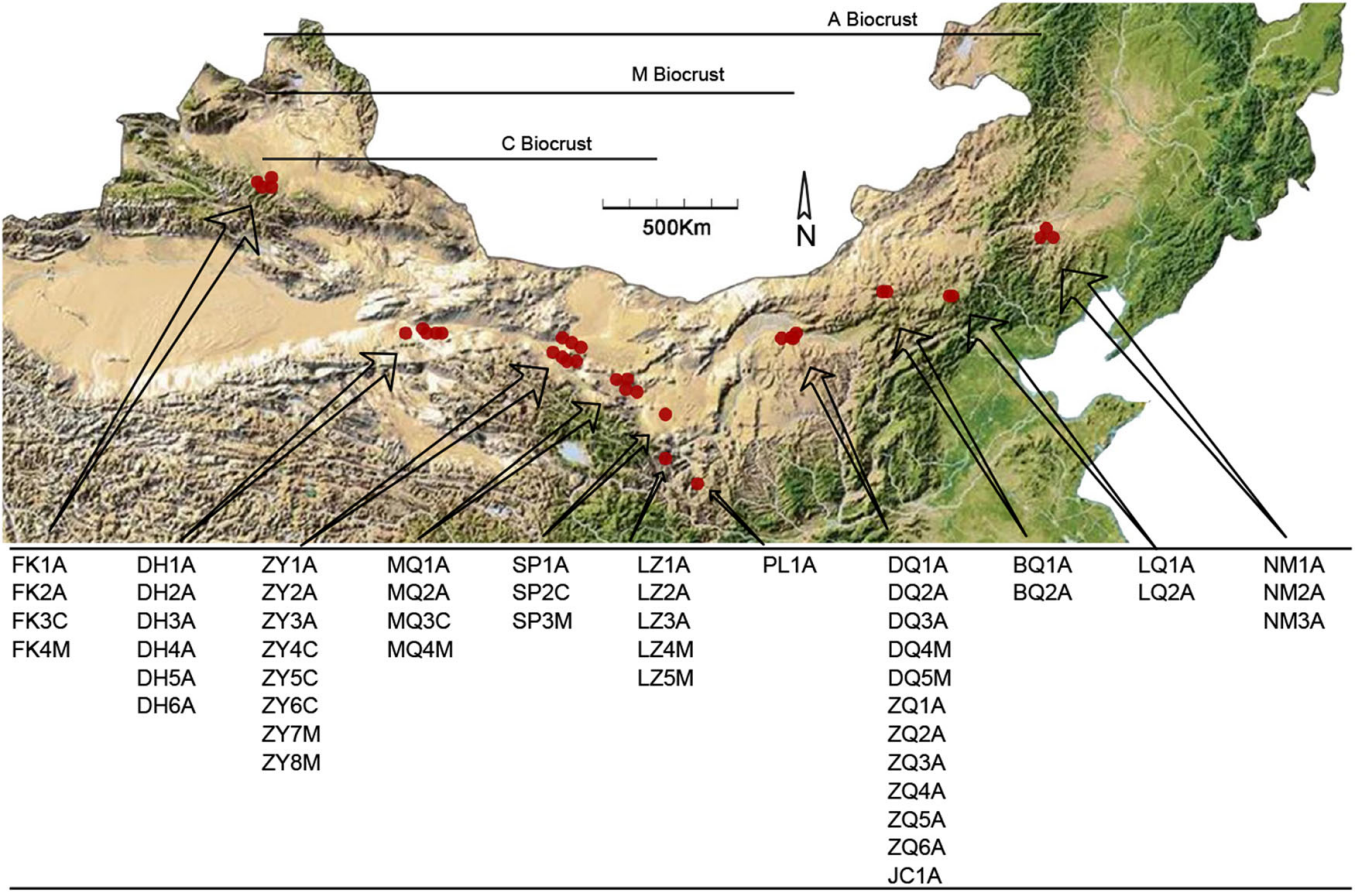
Study area and sampling sites of north China. The biocrusts were sampled and showed in geomorphic map. There were 35 algae-dominated (A) crust sites, 6 lichen-dominated (C) crust sites, and 9 moss-dominated (M) crust sites shown in the figure, and four parallel samples were collected in each sample site. The black horizontal line indicates the geographical span of each biocrust type. Sample site naming rules: the first two letters and the last letter represented place names and biocrust types, respectively. This map is free to use and could be acquired from BIGEMAP (http://www.bigemap.com) and open source geospatial data cloud (http://www.gscloud.cn). And it also is available from the corresponding author on reasonable request.

In northern China, a wide range of sample sites across seven major deserts and the Loess Plateau extended from west (Fukang (45°18′N, 87°97′E) in the Gurbantungut Desert) to east (Naiman (42°98′N, 120°74′E)), and from north (Fukang) to south (Pingliang (35°34′N, 106°6′E)). Four parallel samples were collected from each sample site in September 2017 and 2018, which gave 140A biocrusts, 24C biocrusts, and 36M biocrusts (Fig. 5). We defined the regional scale as where all three successional biocrust existed containing FK, ZY, MQ, and SP sample sites (see Fig. 5). Each sample site had not received any rainfall in the past 72 h and kept 0.2 m away from the shrubs. Biocrusts and apparently attached subsoil were together collected with a shovel and preserved into the sterilized plastic petri-dishes to ensure the integrality, and then carried to the laboratory within 12 h^6^. To ensure the non-redundancy and representativeness of the sequencing results, the macroscopic moss plants (but not their protonemata) were removed from M biocrusts. The original sampling map was acquired from BIGEMAP (http://www.bigemap.com) and open source geospatial data cloud (http://www.gscloud.cn), and then we marked the coordinates of the sample sites.

WC (%), soil texture (%), TH. (mm), chlorophyll *a* (Chl *a*, μg g^−1^), scytonemin (Scyt., unit mg^−1^ fresh weight), extracellular polysaccharide (EPS, mg g^−1^), bacteriochlorophyll *a* (Bchl. *a*, μg g^−1^), and Fv/Fm were determined by previous methods^70,71^.

Each ground and weighed sample was mixed with distilled water at a ratio of 1:5. After mixing and centrifuging thoroughly, part of the supernatant was taken for pH and ORP (mV) measurements with a YSI-pH100 meter (Yellow Springs, Ohio, USA). The other part of the supernatant was filtered and analyzed for ion content (μmol g^−1^) by ion chromatography (Thermo Scientific™ Dionex™ ICS-5000+, Thermo Fisher Scientific, USA) with a C18 column (CNW IC Guard C18 column, CNW Technologies GmbH, Düsseldorf, Germany). The cation and anion concentrations were respectively calibrated using Dionex Six Cation Standard II (K^+^, Ca^2+^, Na^+^, Mg^2+^, and NH_4_^+^) and Dionex Seven Anion Standard II (Cl^−^, SO_4_^2−^, NO_3_^−^, NO_2_^−^, and PO_4_^3−^). The measurement of HCO_3_^−^ and CO_3_^2−^ concentrations adopted acid-base titration. Salinity (μmol g^−1^) was the sum of eight common ion concentrations. Total nitrogen (TN), TP, and total organic carbon (TOC) were measured by previous methods and expressed as g kg^−1^ biocrusts^6^. Meteorological datasets including MAP (mm), AI, MASD (h), mean annual temperature (MAT, °C), WS (m s^−1^), and Alt. (m) were referenced from the National Meteorological Information Center (http://data.cma.cn/). The soil alkaline protease (ALPT, mg d^−1^ g^−1^), soil alkaline phosphatase (ALP, μmol d^−1^ g^−1^), and soil-β-glucosidase (β-GC, μmol d^−1^ g^−1^) activities were determined by extracellular enzyme activity kit procedures (Suzhou Comin Biotechnology Co., Ltd., Suzhou, China). The net photosynthetic rate (Pn, μmol carbon dioxide (CO_2_) m^−2^ s^−1^) and respiratory rate (R, μmol CO_2_ m^−2^ s^−1^) were measured at temperature of 25 °C and photosynthetically active radiation of 250 μE m^−2^ s^−1^ by the Soil Carbon Release Rate Measuring Device (Beijing Yaxinliyi Science and Technology Co., Ltd., Beijing, China).

Total genomic DNA was extracted from biocrust samples using the PowerSoil ®DNA Isolation Kit (Mo Bio, Carlsbad, CA USA). Primers for the 16S rRNA gene (338F/806R, V3–V4 region) and 18S rRNA gene (3NDF/V4_euk_R2) were used for amplification. Sequencing was performed on the Illumina MiSeq PE300 platform (Illumina, San Diego, CA, USA). The acquired sequences were filtered for quality control using standard procedures (Shanghai Majorbio Bio-pharm Technology Co., Ltd., Shanghai, China). OTUs were generated from defined representative sequences, with clustering at 97% similarity. The RDP classifier was used for taxonomic annotation of representative sequences based on an identity threshold of 0.7 in the SILVA database (release 128) for bacteria (16S_bacteria) and eukaryota (18S_eukaryota).

### Statistical analysis

This study investigated microbial communities from biocrust samples of different succession, longitude, latitude, and MAP to compare the assembly processes and geographical distribution patterns. Each of the study angles defined above was partitioned into three groups: successional stages from A to C to M, longitude location from West (E87°–98°) to Mid-longitude (E98°–109°) to East (E109°–120°), latitude location from North (N42°–45.5°) to Mid-latitude (N38.5°–42°) to South (N35°–38.5°), and precipitation from Low (0–150 mm) to Middle (150–300 mm) to High (300–450 mm), characterized by the gradual changes from severe to medium to mild environments, respectively.

To determine the significance of community composition (OTUs) among three group at each study angle, the function betadisper and ANOSIM were respectively used to evaluate dispersion (variances) and similarities among groups with “vegan” package in R^72^. NMDS and ordination was used to visualize Bray–Curtis dissimilarities of communities in “ggplot2” package in R^73^.

We referred to Stegen’s method^14^ to determine the relative importance of ecological processes. To explore the correlation between community structure and environmental variables, mantel test analysis (Spearman) was performed with community structure at phylum level (relative abundance >0.01%) and environmental factors using the mantel function in “vegan” package in R^72^. The correlations (Spearman) between *R*^2^ (mantel test) and the each components of ecological process were used to find environmental variables that mediated the balance between deterministic and stochastic processes at community-level. For the purpose of estimating species co-occurrence across different habitats and regions, we inferred a metacommunity co-occurrence network for the four study angles using OTUs with 99% cumulative abundance of bacterial and eukaryotic communities, respectively, and captured associations based on Spearman correlation relationships with threshold of 0.7 and Bray–Curtis dissimilarity by CoNet (v1.1.1.beta) to deal with noise and outliers^74^.

The R environment (v3.6.2; http://www.r-project.org/) was used for all the statistical analyses.

### Reporting summary

Further information on experimental design is available in the Nature Research Reporting Summary linked to this paper.

## Supplementary information

Supplementary Information

Supplementary Data

Reporting Summary

## Data Availability

The raw sequence data were uploaded on NCBI under BioProject PRJNA640847. Environmental dataset can be publicly accessed on FigShare (10.6084/m9.figshare.13172411.v1). The original map in this study is free to use and could be acquired from BIGEMAP (http://www.bigemap.com) and open source geospatial data cloud (http://www.gscloud.cn). And it also is available from the corresponding author on reasonable request.

## Code availability

R codes can be publicly accessed on FigShare (10.6084/m9.figshare.13172459.v1)
