# Supplementary material for: Biogeographical patterns and mechanisms of microbial community assembly that underlie successional biocrusts across northern China
Source: NPJ Biofilms Microbiomes. 2021 Feb 5;7:15. doi: 10.1038/s41522-021-00188-6 (PMC7864921; doi:10.1038/s41522-021-00188-6)
Supplement: Supplementary file 1 — Supplementary Information [file 41522_2021_188_MOESM1_ESM.pdf]

ORIGNAL ARTICLE

Biogeographical patterns and mechanisms of microbial community assembly that underlie successional biocrusts across northern China

Running title: Biogeographic pattern and mechanism of assembly in biocrusts

Yuanlong Li[1,2], Chunxiang Hu[1*]

[1] *Key Laboratory of Algal Biology, Institute of Hydrobiology, Chinese Academy of Sciences, Wuhan, 430072, China*
[2] *University of Chinese Academy of Sciences, Beijing, 100049, China*

[*]Correspondence：Chunxiang Hu,
Key Laboratory of Algal Biology, Institute of Hydrobiology, Chinese Academy of Sciences (CAS), Wuhan 430072, China
E-mail: cxhu@ihb.ac.cn
Phone/Fax: +86-27-68780866

Supplementary Table 1 Environmental parameters varying along the three gradients for the four study angles. Environmental dataset was transformed by 'standardize'. Linear regression was carried out according to the order of gradients for the four study angles that we defined to find variables with sharp change (red in bold). Abbreviation: Extracellular enzyme activity (EEA), photosynthesis and pigments (P&P). Environmental factors with different properties were separated by yellow and blue.

		Succession (A-C-M)		Longitude (W-M-E)		Latitude (N-M-S)		MAP (L-M-H)	
		Slope	R^2	Slope	R^2	Slope	R^2	Slope	R^2
Soil properties	Sand	-0.32	0.06**	0.60	0.2**	-0.63	0.19**	0.11	0.01
	Silt	0.37	0.08**	-0.48	0.13**	0.63	0.19**	-0.07	0
	Clay	0.04	0	-0.61	0.21**	0.33	0.05**	-0.16	0.02
	TH.	**1.04**	0.66**	-0.35	0.07**	0.40	0.08**	-0.12	0.01
	WC	0.50	0.15**	-0.27	0.04**	0.75	0.27**	0.17	0.02
	pH	0.24	0.03**	-0.56	0.17**	0.70	0.23**	-0.55	0.18**
	ORP	**-0.38**	0.09**	0.32	0.06**	-0.82	0.32**	0.28	0.05**
Nutrition & ions	TP	0.53	0.17**	-0.79	0.34**	0.64	0.19**	-0.28	0.05**
	TN	0.54	0.18**	-0.44	0.1**	0.14	0.01	-0.37	0.08**
	TOC	0.62	0.23**	-0.10	0.01	0.04	0	0.20	0.02*
	NH_4^+	0.22	0.03*	-0.22	0.03*	0.01	0	0.05	0
	PO_4^{3+}	-0.20	0.02*	-0.39	0.08**	-0.01	0	-0.24	0.04**
	EPS	0.74	0.33**	-0.19	0.02*	0.34	0.06**	-0.10	0.01
	NO_3^-	-0.20	0.02*	-0.49	0.13**	-0.06	0	-0.43	0.12**
	Salinity	-0.18	0.02*	-0.53	0.16**	-0.01	0	-0.44	0.12**
	NO_2^-	-0.13	0.01	-0.37	0.08**	0.01	0	-0.31	0.06**
P&P	BChl *a*	0.33	0.07**	-0.08	0	0.08	0	-0.11	0.01
	Scyt.	0.33	0.07**	-0.16	0.01	0.33	0.05**	-0.02	0
	Chl *a*	0.20	0.02*	0.03	0	0.37	0.07**	-0.22	0.03*
	Fv/Fm	0.83	0.42**	0.30	0.05**	0.24	0.03*	0.45	0.13**
	Pg/R	-0.15	0.01	-0.29	0.05**	-0.08	0	-0.27	0.05**
EEA	ALPT	0.42	0.11**	-0.60	0.19**	0.50	0.12**	-0.37	0.08**
	β-GC	0.53	0.17**	0.33	0.06**	-0.20	0.02*	0.17	0.02
	ALP	0.27	0.04**	0.26	0.04**	0.02	0	0.07	0
Microclimate	Alt.	0.34	0.07**	0.07	0	**0.83**	0.33**	-0.05	0
	MAP	-0.06	0	**0.96**	0.51**	-0.16	0.01	**1.19**	0.87**
	MASD	-0.36	0.08**	0.22	0.03*	-0.75	0.27**	-0.22	0.03*
	AI	-0.26	0.04**	**-0.88**	0.43**	0.04	0	**-0.92**	0.52**
	WS	-0.31	0.06**	0.05	0	**-0.96**	0.44**	-0.13	0.01
	MAT	-0.05	0	-0.58	0.18**	0.82	0.33**	-0.07	0

Supplementary Table 2. Comparison of community differences for the four study angles at regional scales. Regional scale contained FK, ZY, MQ, and SP sample sites (see Fig. 5). Community differences were compared based on Bray-Curtis dissimilarity at operational taxonomic unit level, permutation test=999, *<0.05, **<0.01. Limited by the sample distribution in latitude, the three groups here were not equidistant.

OTU level	Succession	Longitude	Latitude	MAP
Bacteria	0.6368**	0.2453**	0.0667	0.2832**
Eukaryota	0.4135**	0.2192**	0.1609**	0.1797**

Supplementary Table 3. The results of derived-ecological processes basing on the four study angles. This results were derived from Table 1 (%). The trend of community assembly included homogeneous assembly (homogeneous selection + homogeneous dispersal) and heterogeneous assembly (heterogeneous selection + dispersal limitation coupled drift). The mechanisms of microbial community assembly included species sorting (homogeneous selection + heterogeneous selection) and dispersal (homogeneous dispersal + dispersal limitation coupled drift). Drift was considered a completely neutral process. A gradient from red to blue shading indicated a value from high to low.

		Succession			Longitude			Latitude			MAP		
		A	C	M	W	M	E	N	M	S	L	M	H
**Bacteria**	Homogeneous	2.51	11.96	4.47	18.36	4.11	1.32	5.72	3.47	7.32	13.83	3.1	2.11
	Heterogeneous	84.38	39.86	82.43	67.91	82.65	67.82	77.78	86.94	78.64	71.43	84.94	89.99
	Neutral	12.98	48.19	13.1	13.74	13.25	30.85	16.51	9.59	14.04	14.74	11.96	7.91
**Eukaryota**	Homogeneous	5.47	10.62	90.31	8.35	22.9	8.54	13.23	6.39	24	8.54	14.59	9.17
	Heterogeneous	73.05	23.81	0	43.65	54.58	71	49.74	74.38	50.11	56.28	59.49	77.86
	Neutral	21.48	65.57	9.68	48.01	22.52	20.46	37.04	19.24	25.89	35.17	25.92	12.97
**Bacteria**	Species sorting	24.88	5.08	21.25	40.31	16.55	18.64	28.36	24.3	20.98	24.03	7.77	30.49
	Disperse	62.01	46.74	65.65	45.96	70.21	50.5	55.14	66.11	64.98	61.23	80.27	61.61
	Neutral	12.98	48.19	13.1	13.74	13.25	30.85	16.51	9.59	14.04	14.74	11.96	7.91
**Eukaryota**	Species sorting	4.45	7.69	34.44	6.17	18.98	4.04	8.04	4.97	19.92	6.05	11.91	6.86
	Disperse	74.07	26.74	55.87	45.83	58.5	75.5	54.93	75.8	54.19	58.77	62.17	80.17
	Neutral	21.48	65.57	9.68	48.01	22.52	20.46	37.04	19.24	25.89	35.17	25.92	12.97

Supplementary Table 4 The number of abundant taxa nodes and rare taxa nodes in the central cluster of the network. The abundant taxa nodes (relative abundance >5%) were showed with clear phylum name. The rare taxa nodes (relative abundance <5%) were showed as bacteria (<5%) or eukaryota (<5%). in Fig. 4 legend. The criteria to define the central cluster was that there were more than five neighbor nodes around one node and less than one distance between nodes. A gradient from red to blue shading indicated a value from high to low.

	Succession			Longitude			Latitude			MAP		
	A	C	M	W	M	E	N	M	S	L	M	H
Actinobacteria	35	17	20	75	59	19	51	64	42	71	39	31
Proteobacteria	37	32	76	70	79	20	98	47	39	69	36	55
Chloroflexi	19	39	59	41	68	12	84	55	51	52	43	54
Cyanobacteria	23	44	23	42	40	59	42	42	68	49	40	31
Bacteroidetes	28	31	16	44	30	23	34	39	16	48	13	28
Ascomycota	4	22	12	14	25	22	21	29	23	34	36	28
Phragmoplastophyta	1	2	5	2	3	6	2	0	3	3	3	5
norank_k__Chloroplastida	2	1	6	14	8	10	11	2	19	1	5	12
<5% Bacteria	36	35	50	112	77	11	63	68	27	103	30	50
<5% Eukaryota	40	34	7	24	18	63	32	36	49	16	34	54

Supplementary Table 5. The correlation between topological characteristics of the co-occurrence network and ecological processes considering all four study angles. n=12, Spearman correlation, *<0.05, **<0.01, significance values in bold.

	Dispersal limitation/Species sorting (in Bacteria)	Dispersal limitation/Species sorting (in Eukaryota)
Num. edge/Num. node	**-.692***	0.007
Max. degree	**-.718****	0.088
clustering coefficient	-0.521	**.581***
network centralization	**-.664***	0.154
network density	**-.641***	0.116
average degree	**-.692***	0.007

Supplementary Table 6 The correlation between environmental factors and ecological processes considering all four study angles. The four study angles included succession, longitude, latitude, and mean annual precipitation (MAP). The Spearman correlation was calculated between R^2 (Mantel test) and ecological processes (n=12), *<0.05, **<0.01. Significance values in bold. The variables in the red box were related to both the bacterial and eukaryotic communities.

		Bacteria			Eukaryota		
		Homogeneous select	Heterogeneous select	Species sorting	Homogeneous select	Heterogeneous select	Species sorting
Soil properties	Sand	0	-0.161	-0.182	0.035	0.145	0.196
	Silt	-0.056	-0.07	-0.161	-0.154	0.477	0.476
	Clay	0.014	0.133	0.28	-0.371	**.617***	-0.427
	TH.	-0.538	-0.287	-0.413	0.343	0.332	0.28
	WC	-0.413	0.21	0.077	-0.091	0.09	**.713****
	pH	**.629***	0.559	**.755****	-0.224	-0.305	-0.301
	ORP	0.503	0.545	**.692***	-0.42	-0.16	0.098
Nutrition & ions	TP	0.357	0.441	**.636***	0.084	-0.348	**-.706***
	TN	0.266	-0.077	-0.042	-0.259	-0.402	0.399
	TOC	0.343	-0.448	-0.392	-0.294	-0.051	-0.091
	NH_4^+	0.266	0.42	0.552	0.448	-0.016	**-.734****
	PO_4^{3+}	-0.399	0.154	0.119	-0.294	0.465	**-.664***
	EPS	-0.063	-0.469	-0.406	-0.231	0.277	0.154
	NO_3^-	0.531	0.294	0.413	**.587***	-0.488	-0.224
	Salinity	0.566	0.413	**.587***	0.175	-0.348	**-.727****
	NO_2^-	0.077	0.329	0.357	0.552	**-.602***	-0.287
P&P	BChl *a*	-0.538	0.07	-0.007	-0.552	**.688***	0.126
	Scyt.	0.231	-0.21	-0.175	-0.392	0.18	-0.14
	Chl *a*	**.608***	-0.413	-0.266	-0.322	0.531	-0.056
	Fv/Fm	0.063	-0.014	0.042	-0.329	**.754****	-0.175
	Pg/R	0.49	-0.175	-0.112	0.322	0.348	-0.301
EEA	ALPT	-0.266	0.112	0.063	-0.448	**.848****	-0.28
	β-GC	0.441	-0.042	0.189	**-.601***	0.449	-0.196
	ALP	**.720****	0.238	0.434	-0.322	-0.043	-0.294
Macroclimate	Alt.	0.252	0.021	0.119	-0.014	0.402	-0.035
	MAP	0.378	0.483	**.643***	**-.664***	**.676***	-0.49
	MASD	0.273	**.720****	**.720****	0.161	**.613***	0.168
	AI	0.315	0.545	**.678***	-0.245	**.621***	**-.615***
	WS	0.042	**.636***	0.566	0.126	**.641***	0.28
	MAT	0.552	0.294	0.441	-0.049	0.172	0.084
