# Supplementary material for: Biogeographical patterns and mechanisms of microbial community assembly that underlie successional biocrusts across northern China
Source: NPJ Biofilms Microbiomes. 2021 Feb 5;7:15. doi: 10.1038/s41522-021-00188-6 (PMC7864921; doi:10.1038/s41522-021-00188-6)
Supplement: Supplementary file 3 — Reporting Summary [file 41522_2021_188_MOESM3_ESM.pdf]

nature research

Corresponding author(s): Chunxiang Hu

Last updated by author(s): Oct 31, 2020

Reporting Summary

Nature Research wishes to improve the reproducibility of the work that we publish. This form provides structure for consistency and transparency in reporting. For further information on Nature Research policies, see our Editorial Policies and the Editorial Policy Checklist.

Statistics

For all statistical analyses, confirm that the following items are present in the figure legend, table legend, main text, or Methods section.

n/a	Confirmed	
☐	☒	The exact sample size (*n*) for each experimental group/condition, given as a discrete number and unit of measurement
☐	☒	A statement on whether measurements were taken from distinct samples or whether the same sample was measured repeatedly
☐	☒	The statistical test(s) used AND whether they are one- or two-sided<br>*Only common tests should be described solely by name; describe more complex techniques in the Methods section.*
☒	☐	A description of all covariates tested
☒	☐	A description of any assumptions or corrections, such as tests of normality and adjustment for multiple comparisons
☒	☐	A full description of the statistical parameters including central tendency (e.g. means) or other basic estimates (e.g. regression coefficient) AND variation (e.g. standard deviation) or associated estimates of uncertainty (e.g. confidence intervals)
☒	☐	For null hypothesis testing, the test statistic (e.g. *F*, *t*, *r*) with confidence intervals, effect sizes, degrees of freedom and *P* value noted<br>*Give P values as exact values whenever suitable.*
☒	☐	For Bayesian analysis, information on the choice of priors and Markov chain Monte Carlo settings
☒	☐	For hierarchical and complex designs, identification of the appropriate level for tests and full reporting of outcomes
☐	☒	Estimates of effect sizes (e.g. Cohen's *d*, Pearson's *r*), indicating how they were calculated

Our web collection on statistics for biologists contains articles on many of the points above.

Software and code

Policy information about availability of computer code

Data collection	Total genomic DNA was extracted from biocrust samples using the PowerSoil ®DNA Isolation Kit (Mo Bio, Carlsbad, CA USA). The acquired sequences were filtered for quality control using standard procedures (Shanghai Majorbio Bio-pharm Technology Co., Ltd., Shanghai, China). The raw sequence data were uploaded on NCBI under BioProject PRJNA640847. Environmental dataset can be publicly accessed on FigShare (https://doi.org/10.6084/m9.figshare.13172411.v1). R codes can be publicly accessed on FigShare (https://doi.org/10.6084/m9.figshare.13172459.v1).
Data analysis	The R environment (v3.6.2; http://www.r-project.org/) was used for all the statistical analyses. We referred to Stegen's method to determine the relative importance of ecological processes. We captured associations based on Spearman correlation relationships (OTU) with threshold of 0.7 and Bray–Curtis dissimilarity by CoNet (v1.1.1.beta) to deal with noise and outliers.

For manuscripts utilizing custom algorithms or software that are central to the research but not yet described in published literature, software must be made available to editors and reviewers. We strongly encourage code deposition in a community repository (e.g. GitHub). See the Nature Research guidelines for submitting code & software for further information.

Data

Policy information about availability of data

All manuscripts must include a data availability statement. This statement should provide the following information, where applicable:

- Accession codes, unique identifiers, or web links for publicly available datasets
- A list of figures that have associated raw data
- A description of any restrictions on data availability

The raw sequence data were uploaded on NCBI under BioProject PRJNA640847. Environmental dataset can be publicly accessed on FigShare (https://doi.org/10.6084/m9.figshare.13172411.v1). R codes can be publicly accessed on FigShare (https://doi.org/10.6084/m9.figshare.13172459.v1)

Field-specific reporting

Please select the one below that is the best fit for your research. If you are not sure, read the appropriate sections before making your selection.

☐ Life sciences ☐ Behavioural & social sciences ☒ Ecological, evolutionary & environmental sciences

For a reference copy of the document with all sections, see nature.com/documents/nr-reporting-summary-flat.pdf

Ecological, evolutionary & environmental sciences study design

All studies must disclose on these points even when the disclosure is negative.

Study description

Different successional biocrusts (alga, lichen, and moss-dominated biocrusts) were collected across the northern China, and assembly of biocrust microbial communities was investigated by high-throughput sequencing combined with measurements of soil properties and microclimate environments.
In northern China, a wide range of sample sites across seven major deserts and the Loess Plateau extended from west (Fukang (45° 18'N, 87°97'E) in the Gurbantungut Desert) to east (Naiman (42°98'N, 120°74'E)), and from north (Fukang) to south (Pingliang (35° 34'N, 106°6'E)). Four parallel samples were collected from each sample site in September 2017 and 2018, which gave 140 A biocrusts, 24 C biocrusts, and 36 M biocrusts.

Research sample

In northern China, a wide range of sample sites across seven major deserts and the Loess Plateau extended from west (Fukang (45° 18'N, 87°97'E) in the Gurbantungut Desert) to east (Naiman (42°98'N, 120°74'E)), and from north (Fukang) to south (Pingliang (35° 34'N, 106°6'E)). Four parallel samples were collected from each sample site in September 2017 and 2018, which gave 140 A biocrusts, 24 C biocrusts, and 36 M biocrusts

Sampling strategy

Each sample site had not received any rainfall in the past 72 h and kept 0.2 m away from the shrubs. Biocrusts and apparently attached subsoil were together collected with a shovel and preserved into the sterilized plastic petri-dishes to ensure the integrality, and then carried to the laboratory as soon as possible.
To ensure the non-redundancy and representativeness of the sequencing results, the macroscopic moss plants (but not their protonemata) were removed from M biocrusts.

Data collection

C.H. conceived and designed the experiments; Y.L. performed the experiments and analysed the data.
Total genomic DNA was extracted from biocrust samples using the PowerSoil ®DNA Isolation Kit (Mo Bio, Carlsbad, CA USA).
Sequencing was performed on the Illumina MiSeq PE300 platform (Illumina, San Diego, CA, USA). The acquired sequences were filtered for quality control using standard procedures (Shanghai Majorbio Bio-pharm Technology Co., Ltd., Shanghai, China).
The raw sequence data were uploaded on NCBI under BioProject PRJNA640847.

Timing and spatial scale

In northern China, a wide range of sample sites across seven major deserts and the Loess Plateau extended from west (Fukang (45° 18'N, 87°97'E) in the Gurbantungut Desert) to east (Naiman (42°98'N, 120°74'E)), and from north (Fukang) to south (Pingliang (35° 34'N, 106°6'E)). Four parallel samples were collected from each sample site in September 2017 and 2018, which gave 140 A biocrusts, 24 C biocrusts, and 36 M biocrusts

Data exclusions

To ensure the non-redundancy and representativeness of the sequencing results, the macroscopic moss plants (but not their protonemata) were removed from M biocrusts.
First, the emergence of macroscopic moss plant was a sign of environmental modification in biocrust, also was a transition to shrub ecosystem.
Second, the microeukaryotic community was studied in both algae (A), lichen (C)-dominated crusts, correspondingly, and it should still be studied in Moss (M)-dominated crust.
Third, due to the large genome size and biomass of the mosses, the amplified sequence of mosses would account for the most part. It also would affected bacterial amplified sequence.
Forth, some researchers removed the macroscopic moss from M crust when studying the photosynthetic biomass (Langhans et al., 2009)

Reproducibility

All results can be repeated using the methods in this study

Randomization

Four parallel samples were collected from each sample site.
There were 140 A biocrusts, 24 C biocrusts, and 36 M biocrusts.
Three successional biocrusts could not be collected synchronously at each sample site. We defined the regional scale as where all three successional biocrust existed containing FK, ZY, MQ, and SP sample sites (see Fig. 5).

Blinding

Blinding was not possible for our research. We use high throughput sequencing technology with high resolution. There were also four study angles involved.

Did the study involve field work? ☒ Yes ☐ No

Field work, collection and transport

Field conditions

In northern China, drylands

Location

In northern China, a wide range of sample sites across seven major deserts and the Loess Plateau extended from west (Fukang (45°

Location	18'N, 87°97'E) in the Gurbantungut Desert) to east (Naiman (42°98'N, 120°74'E)), and from north (Fukang) to south (Pingliang (35° 34'N, 106°6'E)).
Access & import/export	samples were preserved into the sterilized plastic petri-dishes to ensure the integrality, and then carried to the laboratory as soon as possible.
Disturbance	Each sample site had not received any rainfall in the past 72 h and kept 0.2 m away from the shrubs. Biocrusts and apparently attached subsoil were together collected with a shovel and preserved into the sterilized plastic petri-dishes to ensure the integrality, and then carried to the laboratory as soon as possible.

Reporting for specific materials, systems and methods

We require information from authors about some types of materials, experimental systems and methods used in many studies. Here, indicate whether each material, system or method listed is relevant to your study. If you are not sure if a list item applies to your research, read the appropriate section before selecting a response.

Materials & experimental systems

n/a	Involved in the study
☒	☐ Antibodies
☒	☐ Eukaryotic cell lines
☒	☐ Palaeontology and archaeology
☒	☐ Animals and other organisms
☒	☐ Human research participants
☒	☐ Clinical data
☒	☐ Dual use research of concern

Methods

n/a	Involved in the study
☒	☐ ChIP-seq
☒	☐ Flow cytometry
☒	☐ MRI-based neuroimaging
